# Coexisting amyotrophic lateral sclerosis and chorea: A case report and literature review

**DOI:** 10.1097/MD.0000000000032452

**Published:** 2022-12-30

**Authors:** Anni Zhang, Hongbei Xu, Jing Huang, Huilan Gong, Shipeng Guo, Xiaoyang Lei, Dian He

**Affiliations:** a Department of Neurology, Affiliated Hospital of Guizhou Medical University, Guizhou, China.

**Keywords:** amyotrophic lateral sclerosis, case report, chorea, genetic mutation

## Abstract

Amyotrophic lateral sclerosis (ALS) coexisting with chorea is very rare.

**Case report:**

We present the case of a 48-year-old man with ALS and chorea; the diagnostic certainty was high based on clinical examination results. Combining the data from literature, we analyzed the characteristics of patients with ALS and chorea. We found that ALS coexisting with chorea is very rare, but is often hereditary with a genetic mutation. Most patients with ALS and chorea are caused by abnormal amplification of a CAG sequence in the HTT gene, and these patients have a mild course of disease. The FUS, VCP, and SETX genes also have low mutation frequencies in patients with ALS and chorea.

**Conclusion:**

The abnormal amplification of a CAG sequence in the HTT gene in ALS with chorea has an obvious familial genetic tendency, and most patients have a mild disease course.

## 1. Introduction

As a chronic progressive neurodegenerative disease, amyotrophic lateral sclerosis (ALS) leads to upper and lower motor neuron damage, muscle weakness, and atrophy. In rare cases, ALS is accompanied by other extrapyramidal diseases, including classic Parkinson disease (also known as Brait-Fahn-Schwartz disease),^[1]^ Parkinson-dementia syndrome,^[2]^ multisystem atrophy,^[3]^ and progressive supranuclear palsy.^[4]^ Chorea is a hyperkinetic movement disorder characterized by involuntary, transient, random, and irregular contractions.^[5]^ Chorea has a number of different causes, including neurodegenerative diseases, inherited metabolic diseases, autoimmune diseases, and various other impairments that affect basal ganglia function.^[6]^ The classic form of chorea appears in Huntington disease (HD), which is caused by repeated amplification of a CAG sequence in the HTT gene.^[7]^ Caudate nucleus atrophy is the main pathology underlying HD.^[7]^ The prevalence of coexisting ALS and HD is 2–6 cases per billion people.^[8,9]^ Although some cases of ALS complicated by HD have been reported, the clinical and genetic characteristics have not been established. Herein we report a case of a Chinese patient with coexisting ALS and chorea. We also reviewed the literature and summarize the clinical, imaging, genetic, and pathologic characteristics of ALS with chorea to improve clinicians’ understanding of this disease combination, and to facilitate the early diagnosis, treatment, and improved prognosis of these patients.

## 2. Case presentation

### 2.1. Basic information

A 48-year-old man experienced choreiform movements of the head and neck with mild involvement of the extremities (Supplement Video 1, Supplemental Digital Content). He was referred to our hospital at 48 years of age on November 16, 2020. Four months later, he had dysphagia and weakness in the arms and hands. The muscle weakness progressed and atrophy developed in the distal upper extremities in 2021. He sought evaluation in our hospital on September 23, 2021. There was no family history of degenerative disorders. No sexual dysfunction was reported, but the patient complained of irritability. The oculomotor examination was normal. The physical examination revealed chorea, mainly in the neck, shoulders, and distal lower extremities, and mostly on the left side. Chorea was observed throughout the examination at rest or in action, and was exacerbated by movement. He also had a reduced pharyngeal reflex with tongue atrophy and fasciculations. Severe amyotrophy was noted in the thenar and shoulder girdle muscles. Ongoing and chronic denervation changes in the bulbar, cervical, thoracic, and lumbar segments were shown during a needle electromyography (EMG) examination. The upper extremities showed distally pronounced weakness (right/left, Medical Research Council [MRC] grades), as follows: neck flexion 5/5; neck extension 4/5; shoulder abduction 5/5; forearm flexion 5/5; wrist flexion 4/5; wrist extension 4/5; and thumb abduction 4/5. The lower extremity muscle strength was normal. Pathologic brisk deep tendon reflexes (3+) were noted throughout. Bilateral palmomental reflexs, and Hoffman and Babinski signs were positive; the other pathologic reflexes were negative. The remaining neurologic examination findings were unremarkable.

**Figure 1.**
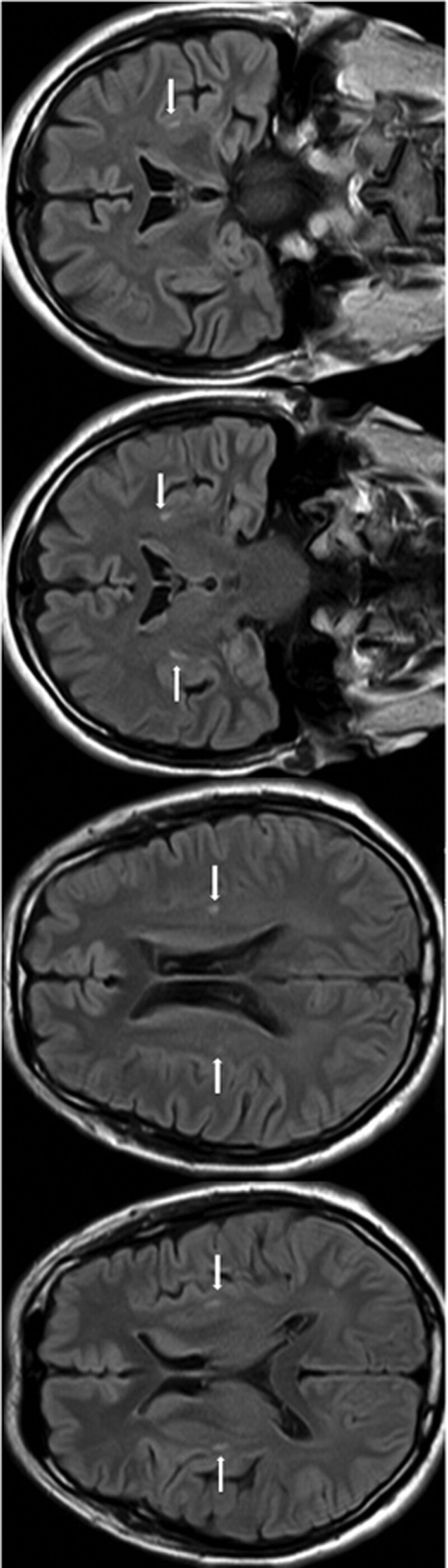
Brain MRI results of the patient on admission. Brain MRI axial FLAIR imaging and coronal FLAIR imaging exhibited bilateral abnormal putamen signals; arrows point at abnormal signals.

Nerve conduction studies showed decreased amplitudes of compound muscle action potentials in the right (4.5 mV; normal > 5 mV) and left median nerves (2.2 mV; normal > 5 mV). Compound muscle action potential amplitudes in the ulnar, tibial, and peroneal nerve studies were normal bilaterally. The amplitudes of sensory nerve action potentials were severely decreased in the right ulnar nerve (2.5 uV; normal > 7.0 uV). Nerve conduction study findings showed a decreased motor conduction amplitude of the right median nerve and decreased motor conduction velocity of the ulnar nerve above the elbow (AE) and below the elbow (BE). The amplitude of sensory conduction in the right ulnar nerve was decreased. EMG showed evidence of diffuse active and chronic denervation in the tongue, left sternocleidomastoid muscle, abductor muscle of the left little finger, bilateral common digital extensor muscles, bilateral T9-paraspinalis muscles, and the right vastus lateralis muscles. Fibrillation potentials were present in the abductor muscle of the left little finger, left common digital extensor muscle, left sternocleidomastoid muscle, and left T9-paraspinalis muscle. Changes in motor unit potentials, including polyphasia and long duration, were observed in all the muscles examined. Findings suggesting unstable neuromuscular transmission (jitter/blocking of polyphasic spikes) in the tongue, sternocleidomastoid muscle, tibialis anterior muscle, and T9-paraspinalis muscle. Therefore, active, or chronic denervation was demonstrated in the bulbar, cervical, thoracic, and lumbar segments. Magnetic resonance imaging studies revealed bilateral abnormal putamen signals (Fig. 1) and slight spondylotic changes in the cervical spine, without signal abnormalities. Cerebral fluorodeoxyglucose positron emission tomography showed no abnormities.

### 2.2. Laboratory test results

The findings from biochemical tests were unremarkable while screening for infections, toxins, and metabolic and systemic autoimmune diseases. Serum and cerebrospinal fluid paraneoplastic antibody assays (CV2, Ma2, Hu, Yo, Ri, and amphiphysin) and autoimmune encephalitis antibodies (CASPR2, GABAb, NMDAR, LGI1, AMPAR1, and AMPAR2) were negative. Next-generation sequencing and dynamic mutation detection (PCR + fragment analysis (capillary electrophoresis) methods were used. The results of genetic testing for HD-related genes (DRPLA, FRDA1, SCA1-2-7-17, VPS13A, JPH3, and the androgen receptor gene) and related genes of familial hereditary amyotrophic lateral sclerosis [FALS] (C9orf72, UBQLN2, FUS, TARDBP, SOD1, VCP, and PGRN) were negative. Gene tests revealed normal CAG expansion with 18 and 19 CAG repeats for each of the 2 alleles of the IT15 huntingtin gene. Serum ceruloplasmin (urinary Cooper) was normal (292 mg/L). His only daughter currently did not exhibit any clinical neurologic symptoms. His parents were deceased, and there was no information was available regarding other family members.

### 2.3. Diagnosis and follow-up

The clinical features, electrodiagnostic features, and laboratory test results suggested that the patient had ALS combined with chorea. The patient was discharged to home on October 1,2021. Oral treatment with haloperidol (2 mg tid) and riluzole (50 mg bid) reduced the frequency of involuntary dance-like movements.

At the follow-up visit in January 2022, there was no significant aggravation of involuntary movements. The patient had clear consciousness, but still had weakness in both upper extremities, difficulty in shrugging, and slurred speech. The distal muscle strength of both upper extremities was grade 4, and the distal muscles of both upper extremities, and interosseous and thenar muscles were atrophied. The supraspinatus muscles were atrophied. The patient did not develop parkinsonism. No psychiatric features or cognitive impairment were noted. The results of the other examinations were unchanged.

### 2.4. Clinical and genetic features of the cases from the literature review

Twenty ALS patients with chorea were identified in the literature search until December 2021^[10–26]^ were collected with our case report, and the clinical and genetic features are summarized in Tables 1 and 2. The gender distribution was nearly equal (10 males and 11 females), and 52.4% (11/21) of the patients had FALS or chorea. Among the 11 patients with a known family history, 2 had FALS, 2 had hereditary chorea, 6 had hereditary HD, and 1 patient had both FALS and hereditary HD. The mean age of onset was 53.6 ± 11.7 years (age range, 30–72 years; male mean age, 53.5 ± 12.1 years; female mean age, 53.6 ± 11.3 years). All 21 patients were clinically characterized by diffuse dance-like movements, and 10 patients had cognitive impairment. Other symptoms, such as eye movement disorders (4 cases), rigidity (2 cases), personality change (1 case), gait disorder (1 case), falls (3 cases), and Parkinson symptoms (1 case), were not prominent. Of the 9 patients with ALS onset followed by chorea, 2 had bulbar onset, 2 had cervical onset, and 2 had lumbar onset; the other 3 are unknown. Among the 12 patients who underwent imaging examinations, 4 had caudate nucleus atrophy, 3 had frontal hypoperfusion, 1 had pyramidal tract atrophy, 1 had cervical spinal cord atrophy, 1 had an abnormal putamen signal shadow, 1 had a white matter abnormal signal, and 1 had no imaging abnormalities. At present, the genetic test results of 14 patients were positive: 11 patients had CAG(HTT) gene mutations; 1 patient had an FUS gene mutation; 1 patient had both VCP and CAG gene mutations; and 1 patient had an SETX gene mutation.

**Table 1:** Summary of cases identified in the literature search.

Case	Reference	Sex	Onset age	Family history	Disease order	Off-time	Duration	Clinical symptoms	ALS initial segment	Imaging findings	Genetic test results	Pathology findings
1	^[10]^	M	66	ALS (mother); neuropsychiatric disorder (maternal uncle)	Chorea→ALS	NA	15 years	Diffuse choreiform movements, cognitive changes, impaired saccadic oculomotor velocity, mild limitation of upwards gaze, dysarthria	NA	Caudate atrophy	HD (CAG = 45)	Severe atrophy of caudate nucleus and putamen; neuronal loss in anterior horns of cervical (upper) and upper lumbar (lower) spinal cord
2	^[11]^	M	56	HD (paternal first cousin), Parkinson disease (mother)	Chorea→ALS	7 months	NA	Gait disturbance, upper limb weakness and wasting, diffuse choreiform movements, behavioral changes	NA	NA	HD (CAG ≥ 40)	NA
3*	^[12]^	F	58	Chorea (paternal grandmother)	ALS→chorea	3 years	11 years	Dysarthria, diffuse choreiform movements, emotional lability, difficulty concentrating, cognitive changes, distal atrophy of upper limbs, fasciculations in all limbs	Cervical	Caudate atrophy	HD (CAG = 46)	Atrophy of neostriatum and spinal anterior horn; antihuntingtin (EM48) and antipolyglutamine (1C2) antibodies in spinal anterior horn cells; TDP-43-positive inclusions in neurons of entire cerebral cortex, including motor cortex
4*,†	^[12]^	F	30	HD (father, paternal grandfather and relatives, sister)	Chorea→ALS	NA	28 years	Diffuse choreiform movements, cognitive impairment, rigidity	NS	Caudate atrophy	HD (CAG = 47)	Atrophy of the neostriatum; severe neuronal loss and gliosis in caudate, spinal anterior horn, and especially cervical and thoracic segments; TDP43 antibody was positive in cortex and spinal cord; glutamine antibody and spinal cord TDP43 antibody inclusion bodies were positive in motor cortex, amygdala, putamen and brain stem
5‡,§	^[12]^	F	50	HD (numerous maternal relatives)	Chorea→ALS	NA	≥16 years	dysarthria and dysphagia, postural instability, involuntary limb and trunk movements, cognitive and personality changes	Bulbar	NA	HD (CAG = 42)	NA
6*	^[12]^	F	48	HD (father)	Simultaneously	0	≥2 years	Dysarthria, tongue atrophy, weakness in all limbs, spasticity	Bulbar	Signal changes in the corticospinal pathways bilaterally from motor cortex to brain stem	HD (CAG = 39)	NA
7*	^[13]^	M	40	N	ALS→chorea	10 years	NA	Weakness of all 4 limbs, dysarthria and dysphagia, choreiform movements affecting the 4 limbs and the face, muscle atrophy, slow ocular saccades	Lumbar	Bilateral hypometabolism in the frontal lobes	N	Motor neuron loss in the brain stem, medulla, pallidum, substantia nigra, and supranuclear centers; intranuclear ubiquitin-positive inclusions in motor neurons
8	^[14]^	F	53	ALS (father, sister)	ALS→chorea	31 months	47 months	Diffuse choreiform movements, weakness and atrophy in the lower limbs	Lumbar	NA	N	Neuron loss in subthalamus, substantia nigra pars compacta, red nucleus, globus pallidus, anterior horn, and spinal cord
9	^[15]^	M	47	Paget disease, HD (maternal relatives), ALS (paternal relatives)	ALS→chorea	6 months	4.5 years	Paget disease, vocal tics, cognitive change, dementia, slow saccades, chorea and profound weakness with widespread muscle atrophy and fasciculations, rigidity and spasticity of all limbs	Bulbar	NA	VCP gene (R155H), HD (CAG = 44)	Focal gliosis in caudate nucleus; bilateral corticospinal tract degeneration and depletion of anterior horn cells; TDP-43-positive inclusions in anterior horn cells, neocortex and hippocampus
10	^[16]^	F	43	N	Chorea→ALS	1 year	2.5 years	Choreiform movements in the limbs, dysphagia, dysarthria, upper leg fasciculations and arm muscle atrophy	Cervical	N	FUS	NA
11	^[17]^	F	72	HD	ALS→chorea	18 months	NA	Involuntary movements of the head and extremities, frequent falls, dysarthria and dysphagia, frontal lobe dysfunction	Bulbar	Mild bifrontal and right temporal hypoperfusion, mildly decreased blood flow in frontal cortex	HD (CAG = 44)	NA
12	^[18]^	M	41	Father and siblings had involuntary movements in the limbs in their 60s	ALS→chorea	1 year	NA	Weakness and atrophy; choreiform movements in face, upper extremities and trunk; cognitive impairment	Cervical	Mild diffuse cortical atrophy	HD (CAG = 46)	NA
13	^[19]^	M	69	N	Simultaneously	0	NA	Diffuse choreiform movements; memory problems; fasciculations of arms, thighs, calves, and upper chest; weakness of limbs; atrophy of upper limbs	NA	Mild periventricular white matter signal changes consistent with microvascular ischemia	HD (CAG = 40)	NA
14	^[20]^	M	64	HD (father)	Chorea→ALS	10 years	NA	Weakness of limbs, atrophy of the right hand intrinsic fasciculations in the distal hands, diffuse choreiform movements, dysphagia	Bulbar	NA	HD (CAG = 39)	NA
15	^[21]^	F	65	NA	ALS→chorea	NA	7 years	Progressive gait disorder, arm/hand weakness, muscle atrophy, dysarthria, diffuse choreiform movements	NA	NA	NA	FUS protein is in the neocortical region
16	^[22]^	F	62	N	NA	NA	NA	Loss of balance and frequent falls. chorea, dystonia, parkinsonism, weakness, atrophy, spasticity	NA	NA	N	NA
17	^[23]^	M	66	N	NA	NA	NA	Dysphagia, deep voice, cognitive impairment, falls, chorei form movements of limbs	NA	Atrophy of caudate nucleus	HD (CAG = 40)	NA
18	^[24]^	F	54	N	Chorea→ALS	NA	NA	Involuntary movements of face and upper limbs, progressive weakness of left arm, scattered fasciculations	Cervical	Slight widespread supratentorial atrophy and marked thinning of the cervical spinal cord predominantly involving the C4–C5 segments	SETX	NA
19	^[25]^	F	55	N	ALS→chorea	22 years	NA	Weakness of upper limbs, atrophy with fasciculations of tongue, dyspnea, choreiform movements in neck and face	NA	NA	NA	NA
20	^[26]^	M	38	N	ALS→chorea	2 years	2.5years	Horizontal gaze palsy, hemiballismus and chorea	NA	NA	NA	Motor neuron loss in the bulbar and cervical motor nuclei
21	This case report	M	48	N	Chorea→ALS	4 months	still alive	Choreiform movements in limbs, dysphagia, fasciculations and atrophy of tongue, weakness and atrophy of upper limbs	Bulbar	Abnormal putamen signal	N	NA

ALS = amyotrophic lateral sclerosis, F = female, HD = Huntington disease, M = male, N = negative, NA = not available.

* Confirmed HD mutation carrier.

† Autopsy-confirmed ALS.

‡ Laboratory-confirmed ALS.

§ Supposed age at when the case was reported.

**Table 2:** Clinical and gene mutation characteristics of 21 patients.

Characteristic	Mean ± SEM/N (%)
Sex	
Male	10 (47.6)
Female	11 (52.4)
Family history	
Positive	11 (52.4)
Negative	9 (42.9)
N/A	1 (4.8)
Age of onset	
Males	53.5 (38–69)
Females	53.6 (30–72)
Onset order	
ALS→chorea	9 (42.9)
Chorea→ALS	8 (38.1)
Simultaneously	2 (9.5)
N/A	2 (9.5)
Mutation	
HTT	11 (52.4)
FUS	1 (4.8)
VCP and CAG	1 (4.8)
SETX	1 (4.8)
negative	4 (19.1)
N/A	3 (14.3)

## 3. Discussion

In the process of studying ALS pathology, it was suggested that ALS is not only a neurodegenerative disease, but also correlates with multisystem involvement.^[27]^ A previous study suggested that ALS patients may also present with atypical symptoms, such as sensory disturbances, dementia, extraocular palsy, and cerebellar and extrapyramidal symptoms.^[28]^ The revised El Escorial criteria define these extremely rare, multisystem involvement ALS types as ALS Plus.^[29]^ The coexistence of ALS or FALS and HD is very rare because the prevalence of both diseases is very low. Therefore, studying ALS with chorea is challenging. Our patient showed remarkable weakness and atrophy of the upper extremities, choreiform movements of the head and neck, dysphagia, and tongue fasciculations and atrophy. Chronic denervation changes in the tongue muscles, extremities, and T9-10 paraspinal muscles were demonstrated on EMG. These findings were suggestive of ALS with chorea and a bulbar onset, followed by upper extremity atrophy and the gradual appearance of diffuse chorea. The clinical manifestations were similar to the clinical manifestations in previous reports.^[10–26]^

Knowledge of ALS with chorea is based on sporadic and heterogeneous cases. The results of the 20 cases of ALS with chorea collected from the literature review^[10–26]^ suggested that ALS with chorea occurs equally in men and women. We must, however, emphasize that this is a rough estimate due to the small sample size. The average onset age of ALS with chorea was approximately 54 years. In ALS patients with chorea, the percentage of patients with a first onset of ALS symptoms was similar to that of patients with a first onset of chorea symptoms, and only 2 patients had an onset of symptoms of ALS and chorea symptoms simultaneously. The survival time of greater than one-half of the patients was > 7 years,^[10,12–16,20,21,25,26]^ which is longer than the survival time of patients with ALS alone (3–5 years),^[27–29]^ suggesting that the disease progression of such rare ALS Plus patients is mild and the survival time is relatively long.

Approximately one-half of ALS patients with chorea have a positive family history, in particular, a family history of HD.^[11,12,17,20]^ In one case, the patient who had genetically confirmed HD subsequently developed rapidly progressive ALS.^[30]^ This suggests that ALS with chorea has an obvious genetic predisposition, especially a family history of HD,^[31]^ which should be considered clinically.

Several possible mechanisms have been raised about motor neuronal loss in HD. It is generally thought that the aggregation of prolonged polyglutamine peptides in neurons lead to abnormally prolonged CAG repeats in the IT15 gene,^[32]^ which may cause cellular dysfunction.^[33]^ Bunina bodies in the anterior horn cells and the prolonged prolonged polyglutamine peptides lead to neuronal dysfunction, or even death in HD patients,^[10,34]^ as occurs in ALS. The extended glutamine polypeptide is a toxic protein encoded by an abnormal repeat CAG sequence in the HTT1 gene.^[35,36]^ The HTT gene mutation is most common in ALS patients with chorea. A previous study showed that 2 patients with an abnormal HTT gene CAG duplication showed basal ganglia atrophy on autopsy, and positive polyglutamine antibody and positive TDP34 antibody inclusion bodies coexisted in the motor cortex, amygdala, putamen, and brainstem.^[12]^

VCP mutations in FALS and sporadic ALS (SALS) have been confirmed. Several neurologic diseases such as frontotemporal dementia (IBMPFD) and inclusion body myopathy (IBM) with Paget disease of bone are caused by VCP mutations. As an autosomal-dominant multisystem degenerative disease, IBMPFD affects the brain, bone, and muscle. IBMPFD is similar to ALS and frontotemporal dementia, and is also characterized by TDP-43-positive, ubiquitinated inclusions in muscles and frontal cortex neurons.^[37]^ In models of HD, mitophagy impairment and neurodegeneration is caused by VCP recruitment to mitochondria.^[38]^ Unlike patients with ALS and HTT gene mutations, patients with ALS and VCP and HTT gene mutations have a history of Paget disease, with obvious ankylosis and damage to upper motor nerves in the cervical and thoracic regions detected on physical examination.^[15]^ Local atrophy of the right hemisphere superior temporal gyrus, anterior central gyrus, and prefrontal region and abnormal gliosis of the caudate nucleus were demonstrated on autopsy. Compared to patients with HTT mutations, patients with VCP and HTT mutations have TDP43-positive inclusion bodies, not only in the spinal cord, but also in the hippocampus.^[15]^ These results suggest that multisystem degenerative diseases caused by VCP gene mutations may also involve a wide range of HTT gene mutations.

Greater than 50 FUS mutations have been identified that cause FUS-mediated toxicity in ALS patients.^[34,39]^ FUS/TLS is closely associated with ALS and frontotemporal lobar degeneration.^[40]^ The coaggregation between mutant huntingtin and FUS/TLS is mediated by a prion-like domain in the N-terminus of FUS/TLS.^[40]^ FUS-mutated ALS with chorea develops at an early age and has a prominent history of leg and arm chorea, but the disease progresses quickly.^[16]^ The more subtle features of FUS-gene-associated ALS with chorea remain to be explored.

Senataxin is encoded by SETX and is considered to be involved in the prevention of DNA damage and in processing noncoding RNAs.^[41]^ Senataxin gene missense mutations cause ataxia with oculomotor apraxia type 2, which leads to cerebellar atrophy and occasional oculomotor apraxia.^[41]^ Heterozygous mutations in SETX are also found to cause dominantly inherited juvenile amyotrophic lateral sclerosis (ALS4).^[41]^ The medulla oblongata and respiratory muscle function are preserved is patients with ALS4 and the course is benign, but distal muscle weakness and atrophy progresses slowly with pyramidal signs.^[42]^ Patients with ALS and chorea with SETX mutations show characteristics different from ALS4, such as late onset, bulbar involvement, and a major proximal distribution of muscular atrophy, accompanied by dance-like movements.^[24]^ Therefore, SETX gene analysis should be performed in patients with ALS and chorea complicated with an elevated AFP level. In a large family identified as ALS with chorea, the I113T SOD1 mutation was confirmed and these cases showed an extreme variability of the disease.^[43]^

In our case report clinical evidence of UMN and LMN signs in the bulbar region were presented, such as dysphagia, tongue atrophy, fasciculations, and positive bilateral palmomental reflexes, whereas electrophysiologic evidence of LMN signs in the bulbar region showed changes in motor unit potentials and unstable neuromuscular transmission (jitter/blocking of polyphasic spikes) in the tongue. Upper motor neuron (UMN) and lower motor neuron (LMN) signs in the cervical segment showed brisk deep tendon reflexes (3+) in the upper extremities, Hoffman sign, and atrophy in the thenar muscles and shoulder girdle muscles. UMN signs with brisk deep tendon reflexes (3+) in the lower extremities and active or chronic denervation in the thoracic segments were clinical and electrophysiologic evidence of UMN and LMN involvement in the thoracic segments. The patient’s condition fulfilled clinically definite ALS according to the 2008 Awaji-Shima criteria.^[44]^

After a careful check of the literature, we found that bilateral symmetric high signal intensity in the putamen on T2-weighted images are associated with Wilson disease (WD).^[45]^ Atrophy of the putamen exists at the time of clinical motor onset in Huntington disease.^[46]^ Interestingly, our case showed abnormal putamen signals, but not WD and HD. ^18^F-FDG PET is capable of showing striatal dysfunction in chorea.^[47–49]^ Therefore, fluorodeoxyglucose positron emission tomography was used in our case to elucidate basal ganglia function.

Huntington disease-like 1 (HDL1) is a rare presentation of autosomal dominant familial prion disease, but there was no family history of HDL1 in our case. The age of onset in our case was > 45 years, and the rate of progression was slow. We did not find a case report of HDL1 coexisting with ALS. So, we did not consider the diagnosis of WD. It was necessary to do PRNP expansions of the repetitive tracts, but laboratory conditions do not allow this genetic testing. Further genetic investigation, including SETX and other chorea-related genes (i.e., ADCY5, PRNP, and VPS13A) are needed, but are lacking due to limitations of laboratory conditions. Indeed, these are the limitations of the current study.

## 4. Conclusion

This paper reports the case of a patient with ALS complicated by chorea, which is a rare type of ALS. No mutation was found in genetic screening, and the etiology is still unclear and needs further exploration.

Based on the literature review, the genetic association of ALS with chorea was strong. ALS with chorea can be caused by abnormal amplification of a CAG sequence in the HTT gene and FUS, VCP, and SETX mutations. The abnormal amplification of a CAG sequence in the HTT gene in ALS with chorea has an obvious familial genetic tendency, and most patients have a mild disease course. The FUS, VCP, and SETX genes have low mutation frequencies in ALS with chorea; however, the more subtle features of ALS with chorea due to mutations in these genes need to be further explored.

## Author contributions

All the authors have participated in the clinical data collection and analysis. AZ and HX did the literature review and analysis, and wrote the 1^st^ draft of the manuscript. JH, HG, and SG validated the data. XL and DH did a critical review and revisions on the manuscript draft. All the authors have approved the final draft for submission.

**Conceptualization:** Anni Zhang, Dian He.

**Data curation:** Anni Zhang, Hongbei Xu, Jing Huang, Xiaoyang Lie.

**Formal analysis:** Hongbei Xu, Huilan Gong.

**Investigation:** Jing Huang, Huilan Gong, Shipeng Guo, Xiaoyang Lie.

**Methodology:** Jing Huang, Huilan Gong, Shipeng Guo.

**Project administration:** Shipeng Guo, Dian He.

**Supervision:** Dian He.

**Validation:** Dian He.

**Writing—original draft:** Anni Zhang, Hongbei Xu.

**Writing—review and editing:** Jing Huang, Huilan Gong, Shipeng Guo, Dian He.
